# Supplementary material for: Malnutrition Screening Tools Are Not Sensitive Enough to Identify Older Hospital Patients with Malnutrition
Source: Nutrients. 2023 Dec 17;15(24):5126. doi: 10.3390/nu15245126 (PMC10745606; doi:10.3390/nu15245126)
Supplement: Supplementary file 1 [file nutrients-15-05126-s001.zip › nutrients-2735771-supplementary.pdf]

Supplementary File S1.

Table S1. Concurrent validity of the SNAQ, MUST, MST, MNA-SF and PG-SGA on malnutrition (A) and moderate or risk of malnutrition (B) against the GLIM criteria in patients aged <70 years.

	**SNAQ** (n=186)		**MUST** (n=186)		**MST** (n=186)	**MNA-SF** (n=186)		**PGSGA-SF** (n=70)	
	A (≥3)	B (≥2)	A (≥2)	B (≥1)	A (≥2)	A (≥3)	B (≥2)	A (≥2)	B (≥9)
False positive, n	1	9	3	5	6	3	44	16	22
False negative, n	25	16	36	12	24	46	13	8	2
Sensitivity, %	66	78	51	84	68	38	82	70	93
Specificity, %	99	92	97	96	95	97	61	63	49
PPV, %	98	87	93	93	89	90	58	54	53
NPV, %	82	87	85	90	82	70	84	77	91
Cohen's Kappa	0.69	0.72	0.53	0.81	0.65	0.39	0.40	0.31	0.36
McNemar	p<0.001	p=0.230	p<0.001	p=0.143	p=0.003	p<0.001	p<0.001	p=0.152	p<0.001

A: malnutrition (cut-off point); B: moderate or risk of malnutrition (cut-off point); PPV: Positive Predictive Value; NPV: Negative Predictive value; SNAQ: Short Nutritional Assessment Questionnaire; MUST: Malnutrition Universal Screening Tool; MST: Malnutrition Screening Tool; MNA-SF: Mini Nutritional Assessment – Short Form; PG-SGA-SF: Patient Generated – Subjective Global Assessment – Short Form; GLIM: Global Leadership Initiative on Malnutrition

Table S2. Concurrent validity of the SNAQ, MUST, MST, MNA-SF and PG-SGA on malnutrition (A) and moderate or risk of malnutrition (B) against the GLIM criteria in patients aged ≥70 years.

	**SNAQ** (n=170)		**MUST** (n=170)		**MST** (n=170)	**MNA-SF** (n=170)		**PGSGA-SF** (n=56)	
	A (≥3)	B (≥2)	A (≥2)	B (≥1)	A (≥2)	A (≤7)	B (≤11)	A (≥9)	B (≥9)
False positive, n	4	8	4	6	10	2	35	11	16
False negative, n	40	34	53	20	29	55	8	10	4
Sensitivity, %	46	54	27	73	61	26	89	66	86
Specificity, %	96	92	96	94	90	98	64	59	41
PPV, %	90	83	83	90	82	91	65	63	61
NPV, %	70	72	63	82	75	63	88	62	73
Cohen's Kappa	0.44	0.48	0.25	0.68	0.52	0.26	0.51	0.25	0.27
McNemar	p<0.001	p<0.001	p<0.001	p=0.009	p=0.003	p<0.001	p<0.001	p=1.000	p=0.012

A: malnutrition (cut-off point); B: moderate or risk of malnutrition (cut-off point); PPV: Positive Predictive Value; NPV: Negative Predictive value; SNAQ: Short Nutritional Assessment Questionnaire; MUST: Malnutrition Universal Screening Tool; MST: Malnutrition Screening Tool; MNA-SF: Mini Nutritional Assessment – Short Form; PG-SGA-SF: Patient Generated – Subjective Global Assessment – Short Form; GLIM: Global Leadership Initiative on Malnutrition

Supplementary File S2. Prevalence of malnutrition and concurrent validity of screening tools against the GLIM criteria based on the Appendicular Skeletal Mass Index (ASMI) (Table 1 and 2) or Skeletal Mass Index (SMI) (Table 3 and 4) threshold for low muscle mass.

Table S3. Prevalence of confirmed malnutrition based on the Appendicular Skeletal Mass Index (ASMI) threshold.

	**n**	**Positive result, n (%)**
GLIM	356	187 (53)
Phenotypic criteria	356	197 (55)
Weight loss	356	113 (32)
Low BMI	356	59 (17)
Low muscle mass	356	151 (42)
Etiologic criteria	356	330 (93)
Reduced intake	356	251 (71)
Inflammation	356	294 (83)

Table S4. Concurrent validity of the SNAQ, MUST, MST, MNA-SF and PG-SGA against the GLIM criteria based on Appendicular Skeletal Mass Index (ASMI) threshold.

	**SNAQ** (n=356)		**MUST** (n=355)		**MST** (n=356)	**MNA-SF** (n=356)		**PGSGA-SF** (n=126)	
	A (≥3)	B (≥2)	A (≥2)	B (≥1)	A (≥2)	A (≥3)	B (≥2)	A (≥2)	B (≥1)
False positive, n	3	10	6	10	12	5	62	21	31
False negative, n	102	82	127	70	88	140	43	21	8
Sensitivity, %	46	56	32	62	53	25	77	68	88
Specificity, %	98	94	96	69	93	97	63	66	49
PPV, %	97	91	91	92	89	90	70	68	65
NPV, %	62	66	56	69	64	54	71	76	79
Cohen's Kappa	0.43	0.49g	0.27	0.56	0.45	0.21	0.41	0.33	0.37
McNemar	p<0.001	p<0.001	p<0.001	p<0.001	p<0.001	p<0.001	p=0.078	p=0.233	p<0.001

A: malnutrition (cut-off point); B: risk of/moderate malnutrition (cut-off point); PPV: Positive Predictive Value; NPV: Negative Predictive value; SNAQ: Short Nutritional Assessment Questionnaire; MUST: Malnutrition Universal Screening Tool; MST: Malnutrition Screening Tool; MNA-SF: Mini Nutritional Assessment – Short Form; PG-SGA-SF: Patient Generated – Subjective Global Assessment – Short Form; GLIM: Global Leadership Initiative on Malnutrition

Table S5. Prevalence of malnutrition based on the Skeletal Mass Index (SMI) threshold.

	**n**	**Positive result, n (%)**
GLIM	356	148 (44)
Phenotypic criteria	356	163 (46)
Weight loss	356	113 (32)
Low BMI	356	59 (17)
Low muscle mass	356	77 (22)
Etiologic criteria	356	330 (93)
Reduced intake	356	251 (71)
Inflammation	356	294 (83)

Table S6. Concurrent validity of the SNAQ, MUST, MST, MNA-SF and PG-SGA against the GLIM criteria based on Skeletal Mass Index (SMI) threshold.

	**SNAQ** (n=356)		**MUST** (n=355)		**MST** (n=356)	**MNA-SF** (n=356)		**PGSGA-SF** (n=126)	
	A (≥3)	B (≥2)	A (≥2)	B (≥1)	A (≥2)	A (≥3)	B (≥2)	A (≥2)	B (≥1)
False positive, n	4	15	7	11	17	6	75	26	37
False negative, n	72	56	97	40	62	110	25	19	7
Sensitivity, %	54	64	37	74	60	30	84	67	88

Specificity, %	98	93	97	95	92	97	63	62	46
PPV, %	96	87	89	91	85	89	64	60	58
NPV, %	73	77	67	83	75	64	83	69	82
Cohen's Kappa	0.55	0.58	0.36	0.70	0.54	0.29	0.45	0.29	0.32
McNemar	p<0.001	p<0.001	p<0.001	p<0.001	p<0.001	p=1.000	p<0.001	p=0.371	p<0.001

A: malnutrition (cut-off point); B: risk of/moderate malnutrition (cut-off point); PPV: Positive Predictive Value; NPV: Negative Predictive value; SNAQ: Short Nutritional Assessment Questionnaire; MUST: Malnutrition Universal Screening Tool; MST: Malnutrition Screening Tool; MNA-SF: Mini Nutritional Assessment – Short Form; PG-SGA-SF: Patient Generated – Subjective Global Assessment – Short Form; GLIM: Global Leadership Initiative on Malnutrition
